# Agrin Promotes Non-Small Cell Lung Cancer Progression and Stimulates Regulatory T Cells *via* Increasing IL-6 Secretion Through PI3K/AKT Pathway

**DOI:** 10.3389/fonc.2021.804418

**Published:** 2022-01-17

**Authors:** Linzhi Han, Hongjie Shi, Shijing Ma, Yuan Luo, Wenjie Sun, Shuying Li, Nannan Zhang, Xueping Jiang, Yanping Gao, Zhengrong Huang, Conghua Xie, Yan Gong

**Affiliations:** ^1^ Department of Radiation and Medical Oncology, Zhongnan Hospital of Wuhan University, Wuhan, China; ^2^ Department of Thoracic and Cardiovascular Surgery, Zhongnan Hospital of Wuhan University, Wuhan, China; ^3^ Department of Biological Repositories, Zhongnan Hospital of Wuhan University, Wuhan, China; ^4^ Tumor Precision Diagnosis and Treatment Technology and Translational Medicine, Hubei Engineering Research Center, Zhongnan Hospital of Wuhan University, Wuhan, China; ^5^ Hubei Key Laboratory of Tumor Biological Behaviors, Hubei Cancer Clinical Study Center, Zhongnan Hospital of Wuhan University, Wuhan, China

**Keywords:** agrin, non-small cell lung cancer, regulatory T cell, interleukin-6, immunosuppression

## Abstract

Non-small cell lung cancer (NSCLC) has high mortality rates worldwide. Agrin contributes to immune synapse information and is involved in tumor metastasis. However, its roles in NSCLC and tumor immune microenvironment remain unclear. This study examined the effects and the underlying mechanisms of Agrin in NSCLC and tumor-infiltrated immune cells. Clinical tissue samples were used to confirm the bioinformatic predictions. NSCLC cells were used to investigate the effects of Agrin on cell cycle and proliferation, as well as invasion and migration. Tumor xenograft mouse model was used to confirm the effects of Agrin on NSCLC growth and tumor-infiltrated regulatory T cells (Tregs) *in vivo*. Agrin levels in NSCLC cells were closely related to tumor progression and metastasis, and its function was enriched in the PI3K/AKT pathway. *In vitro* assays demonstrated that Agrin knockdown suppressed NSCLC cell proliferation and metastasis, while PI3K/AKT activators reversed the inhibitory effects of Agrin deficiency on NSCLC cell behaviors. Agrin expression was negatively associated with immunotherapy responses in NSCLC patients. Agrin knockdown suppressed Tregs, as well as interleukin (IL)-6 expression and secretion, while PI3K/AKT activators and exogenous IL-6 rescued the inhibitory effects. In the mouse model, Agrin downregulation alleviated NSCLC cell growth and Treg infiltration *in vivo*. Our results indicated that Agrin promotes tumor cell growth and Treg infiltration *via* increasing IL-6 expression and secretion through PI3K/AKT pathway in NSCLC. Our studies suggested Agrin as a therapeutically potential target to increase the efficacy of immunotherapy in NSCLC patients.

## Introduction

Lung cancer has the leading occurrence (11.6% of the total cases) and remains the most lethal one (18.4% of the total deaths) all over the world (1). Non-small cell lung cancer (NSCLC) is the major histologic subtype, accounting for approximately 85% lung cancer (2). Despite the improvements in both traditional and novel treatments for NSCLC patients, only partial patients could benefit from these therapies, and the survival rates of the majority remain poor (3, 4).

Agrin is a glycosylated proteoglycan protein involved in the development of neuromuscular junctions (5). Recent studies show that Agrin plays important roles in cancers and immune system. Agrin was reported to be upregulated in various cancers than the adjacent normal tissues (6–9). Further studies found that Agrin upregulation enhanced tumorigenesis and metastasis *via* activating focal adhesion kinase and mitogen-activated protein kinase signaling pathways (6, 10). Moreover, Agrin was reported to activate T cells and facilitate immune connection formation between T cells and target cells (11, 12). Furthermore, Agrin was highly expressed on the membrane of primary T cells and involved in autoimmune disease progression (13). These results suggested that Agrin modulated cancer development and tumor immune microenvironment (TIME), which might be a potential target for tumor immunotherapy. Although Agrin was highly expressed in NSCLC and associated with worse survival, the specific roles of Agrin in NSCLC and TIME are still to be investigated (14).

In our study, we found that Agrin enhanced NSCLC development and regulated tumor-associated regulatory T cell (Treg) infiltration *via* enhancing interleukin-6 (IL-6) expression and secretion through PI3K/AKT pathway, suggesting that Agrin acted as an oncogene and augmented Tregs in NSCLC immune microenvironment. Our findings provided Agrin as a predictive factor of therapeutical approaches for NSCLC patients.

## Materials and Methods

### Data Collection and Preprocessing

RNA-sequencing data (FPKM values) from 33 types of cancers were obtained from the Cancer Genome Atlas (TCGA; https://portal.gdc.cancer.gov). Among them, 510 NSCLC and 59 adjacent normal tissue samples were used for differential expression analysis of Agrin, survival analysis and pathway enrichment analysis. According to Agrin expression levels, 510 NSCLC samples were divided into the high- and low-expression groups. Limma R package was used to analyze the differentially expressed genes, which were regarded as the genes co-expressed with Agrin. These genes were then imported into Metascape (http://metascape.org) for pathway enrichment analysis subsequently. Tumor Immune Estimation Resource (TIMER; cistrome.shinyapps.io/timer) was used to identify tumor-infiltrated immune cells (15). Tumor Immune Dysfunction and Exclusion (TIDE; tide.dfci.harva rd.edu) was used to predict tumor immune evasion, as well as immune checkpoint inhibitor responses (16, 17).

### Clinical Samples

A total of 21 NSCLC samples and their paired normal lung tissues were obtained from Zhongnan Hospital of Wuhan University (Wuhan, China) from January 2018 to April 2020. None of these patients received chemotherapy or radiotherapy before surgery. Patients**’** clinical characteristics were shown in **Table 1**. The studies involving human participants were reviewed and approved by the Institutional Review Board at Zhongnan Hospital of Wuhan University. The patients/participants provided their written informed consent to participate in this study. No potentially identifiable human images or data is presented in this study.

**Table 1:** Patient characteristics.

Variable	Low Agrin expression (n = 10)	High Agrin expression (n = 11)	Total	p-value^*^
			(n = 21)	
Gender				**0.048**
Female	8	3	11	
Male	2	8	10	
Age				0.08
< 65 y	7	3	10	
≥ 65 y	3	8	11	
Smoking				
Yes	2	8	10	**0.048**
No	8	3	11	
Stage				0.39
I	5	8	13	
II	1	2	3	
III	3	1	4	
IV	1	0	1	
T				0.56
T1	4	5	7	
T2	5	6	12	
T4	1	0		
N				0.75
N0	6	7	13	
N1	1	2	3	
N2	3	2	5	
M				0.9
M0	9	11	18	
M1	1	0	1	
Lymph_node_status				0.88
metastasis	3	4	7	
No-metastasis	7	7	14	
Tumor size				0.66
<3.5 cm	7	6	13	
≥3.5 cm	3	5	8	

The bold p-values indicate the statistically significant difference.

### Mice

BALB/c nude mice (5-6 weeks) were purchased from the Vital River Laboratories (Beijing, China). NSCLC H1975 cells (1 × 10^6^ cells per 100 μl) were injected subcutaneously into the right armpits of the mice. Human peripheral blood mononuclear cells (PBMCs, 5 × 10^6^ cells per mouse) were injected subcutaneously into the same place of right armpits 7 days later. The sizes of tumors were measured with digital vernier calipers every 3 days. The volumes of tumors were calculated: volume = (length × width^2^)/2. Mice were sacrificed 45 days after cell inoculation, and tumor tissues were collected and measured for weight and volume. The animal study was reviewed and approved by the Institutional Animal Care and Use Committee at Zhongnan Hospital of Wuhan University.

### Cells

Human NSCLC cell lines (H1299, H1975, H460, A549, PC9), Jurkat T and THP-1 cells were purchased from the Type Culture Collection of the Chinese Academy of Sciences (Shanghai, China). Human bronchial epithelium cell line (BEAS-2B) was purchased from Guangzhou Cellcook Biotech. NSCLC, Jurkat T and THP-1 cells were cultured in RPMI-1640 (Hyclone, USA), while BEAS-2B cells were cultured in DMEM (Gibco, USA), supplemented with 10% fetal bovine serum (FBS, Hyclone). Human PBMCs were collected from healthy donors with informed consent and ethical approval. Monocytes and lymphocytes were harvested using lymphocyte separation medium (Dakewe, China) according to manufacturer’s recommendations.

### RNA Interference

Agrin was downregulated with siRNA-1 (sense: 5’- GCCUGCAAAUCUCUAUCCATT -3’; antisense: 5’- UGGAUAGAGAUUUGCAGGCTT -3’), siRNA-2 (sense: 5’-CCUUUGUCGAGUACCUCAATT -3’; antisense: 5’-UUGAGGUACUCGACAAAGGTT -3’) using jetPRIME^®^ transfection reagent (Polyplus-transfection SA, France) following the manufacturer’s instructions. Lentiviral-mediated shRNAs (GenePharma, China) were used for stable Agrin downregulation, and puromycin (Cayman, USA) was used to select cells at a final concentration of 4 μg/ml.

### Quantitative Real-Time PCR

Total RNAs were obtained using TRIzol (Vazyme, China). RNA (1 μg) was reversely transcribed to cDNA using the HiScript^®^ Q RT SuperMix kit (Vazyme). Quantitative PCR (qPCR) was performed using the ChamQTM SYBR^®^ qPCR Master Mix (Vazyme) on the BIO-RAD CFX96. The relative expression levels of mRNAs were assessed by the 2−ΔΔCt method. The primers are listed in **Table S1**.

### Immunoblotting

Total proteins were extracted using RIPA cell lysis buffer (Beyotime, China) containing phosphatase and protease inhibitors (Sigma, USA). After centrifugated at 13,000 rpm at 4°C for 10 min, supernatants were obtained and boiled with 5 × SDS loading buffer for 10 min. Bicinchoninic acid system (Beyotime) was used to detect protein concentrations. The proteins were separated by 10% SDS-PAGE gels and transferred onto PVDF membranes (Merck). The membranes were blocked with 5% non-fat milk for 90 min, and then incubated with specific primary antibodies at 4°C overnight. The primary antibodies are presented in **Table S2**. After washing, the membranes were incubated with HRP-conjugated antibodies at room temperature for 2 hours. After washing, the proteins were exposed to ECL developer (Aspen) and analyzed by Bio-Rad Image Lab software.

### Immunofluorescence

The cells on 24 × 24 mm glass slides were fixed with 4% paraformaldehyde (PFA, Sangon, China) and permeabilized with 0.5% Triton X-100 (Beyotime) for 15 min. After washing and blocking with 5% bovine serum albumin, the cells were incubated with anti-Ki67 antibodies (1:200) at 4°C overnight. Secondary antibodies (1:200) were applied for 1 hour. Nuclei were stained with DAPI (Sigma), and images were obtained by fluorescent microscope (Olympus, Japan).

### Colony Formation and Cell Proliferation Assays

For colony formation assay, NSCLC cells were cultured in 6-well plates for 2 weeks. The cells were then fixed with 4% PFA, and stained with 0.5% crystal violet (Beyotime). After washing, the numbers of colonies were counted under microscope. For cell proliferation assay, NSCLC cells were seeded into 96-well plates. Cell viability was measured by the CCK-8 reagent kit (Vazyme). The optical density values were estimated at 450 nm everyday using a multimodal plate reader (Molecular Devices, USA).

### Modified Boyden Chamber Assay

NSCLC cells were suspended in RPMI-1640 with 1% FBS and planted into the upper chamber, while RPMI-1640 with 10% FBS was added to the lower chamber. After incubation for 24 hours, the filters were fixed with 4% PFA, and then stained with 0.5% crystal violet. Cells in the upper surface of the chamber were wiped using a cotton swab. Cells at × 100 magnification were counted and photographed. For the invasion assay, the upper chambers were pre-coated with Matrigel (BD, USA, dilution 1:40) before inoculation.

### Wound Healing Assay

NSCLC cells were seeded into 6-well plates until full confluence. After scratched with a 1 ml pipette tip, the migration rates were assessed: wound closure (%) = (distance of initial scratched - distance of final imaged without cells)/distance of initial scratched 48 hours later.

### Enzyme-Linked Immunosorbent Assay (ELISA)

The supernatants were collected from H1299 and H1975 cells 48 hours after transfection. The levels of IL-6 were measured by ELISA kit (Bioswamp, China). The optical density 450 values were determined using SpectraMax^®^ Absorbance Reader.

### Flow Cytometry

Single-cell suspensions of NSCLC cells or PBMCs were stained with the cell cycle staining kit (Beyotime), FITC anti-human CD4, APC anti-human CD25, or PE anti-human Foxp3 antibodies (Biolegend). The stained cells were washed and incubated with 1 ml intracellular fixation buffer at room temperature for 60 min. Permeabilization buffer (2 ml) were then added into each tube, and the cells were centrifuged at 1,500 rpm for 7 min. The supernatants were removed, and single-cell suspensions were analyzed by flow cytometry (Beckman, China).

### Immunohistochemistry (IHC)

Tissue samples were fixed with 4% PFA and embedded in paraffin. After cut into 4 μm slides, deparaffinization, rehydration and blocking were performed in steps, followed by heat-induced antigen retrieval. Next, primary antibodies against Agrin (1:100, Zen), pAKT (1:100, Abclonal), Ki-67 (1:1000, Abcam), IL-6 (1:100, Abclonal), Foxp3 (1:100, Abcam) and CD8 (1:100, Abcam) were used to incubate the slides at 4°C for 12 hours. Consequently, corresponding secondary antibodies were used to incubate the slides at 37°C for 1 hour. Finally, the slides were stained with hematoxylin and then visualized on the light microscopy (Olympus, Japan).

### Statistical Analysis

Data in this study were analyzed with Student’s t-test and one-way ANOVA. All results were presented as Mean ± standard error or mean ± standard deviation (SD). P < 0.05 was defined as statistically significance.

## Results

### Agrin Is Upregulated in NSCLC and Associated With Worse Survival

**Figure 1:**
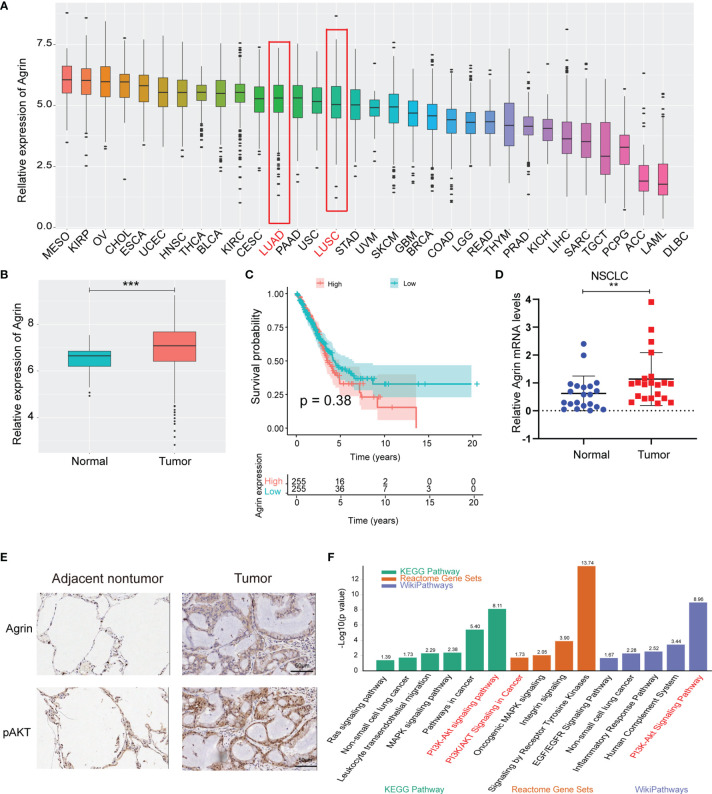
Agrin was upregulated in NSCLC and associated with worse survival. **(A)** Agrin expression landscape in 33 types of cancer. **(B)** Differential expression of Agrin in NSCLC and adjacent normal samples. **(C)** Survival analysis for Agrin in NSCLC patients. **(D)** Differential expression of Agrin in 21 pairs of NSCLC and adjacent tissues. Agrin was significantly upregulated in NSCLC patients. **(E)** Representative IHC images of Agrin and pAKT staining in the clinical tumor and adjacent normal tissues. **(F)** Enrichment analysis of 3 databases (KEGG Pathway, Reactome Gene Sets, Wiki Pathways). **P < 0.01; ***P < 0.001. Student’s t-test.

The landscape map demonstrated Agrin expression levels in 33 types of cancers (**Figure 1A**). As the most lethal tumor, lung cancer was selected to be further analyzed. Agrin was highly expressed in both lung adenocarcinoma and squamous cell carcinoma. The expression levels of Agrin were higher in the NSCLC tumor samples (510 cases) than the adjacent normal tissues (59 cases, *P* < 0.001, **Figure 1B**). The patients with higher Agrin levels had lower long-term survival rates (**Figure 1C**). Moreover, 21 pairs of NSCLC and non-tumor lung tissues were collected from our hospital. Consistently, Agrin was significantly upregulated in tumors compared with the adjacent normal tissues (**Figure 1D**). The IHC results indicated that Agrin and pAKT expression were obviously higher in tumor than adjacent normal tissues (**Figure 1E**). Enrichment analysis also showed that Agrin was significantly associated with the PI3K/AKT pathway (**Figure 1F**).

### Agrin Downregulation Suppresses NSCLC Cell Proliferation and Induces Cell Cycle Arrest *via* Inhibiting PI3K/AKT Pathway

**Figure 2:**
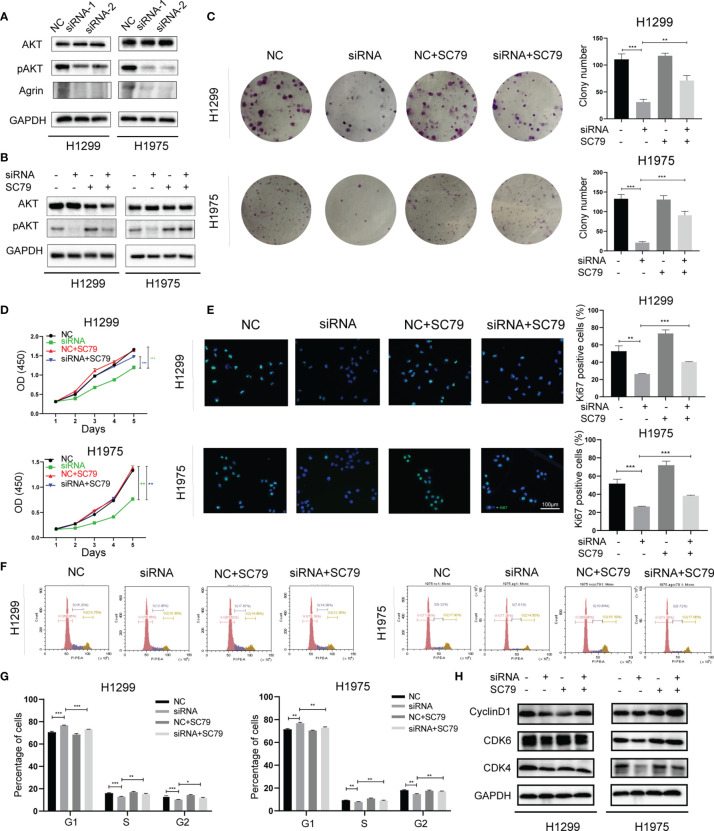
Agrin downregulation suppressed NSCLC cell proliferation and induced cell cycle arrest *via* inhibiting PI3K/AKT pathway. **(A, B)** Agrin knockdown inhibited AKT phosphorylation in H1299 and H1975 cells. **(C)** SC79 (5 μg/ml) rescued colony formation inhibited by Agrin deficiency in NSCLC cells. **(D)** OD values of CCK-8 assay in NSCLC cells treated with Agrin siRNA and SC79. **(E)** Representative images of Ki-67 staining in NSCLC cells treated with Agrin siRNA and SC79. The images were taken at 400X magnification. Scale bar, 100 μm. **(F, G)** SC79 reversed G1 arrest induced by Agrin deficiency in NSCLC cells. **(H)** Representative immunoblots of CyclinD1 and CDK4/6. N = 3; *P < 0.05; **P < 0.01; ***P < 0.001.

Consistent with clinical samples, Agrin was also upregulated in NSCLC cells compared with regular lung epithelial cells (**Figure S1A**). H1299 and H1975 cells with high Agrin expression were transfected with specific siRNAs targeting Agrin, and the downregulation of Agrin was confirmed by PCR (**Figures S1B, C**). Immunoblotting results indicated that Agrin downregulation inhibited AKT phosphorylation, and that AKT activator SC79 restored this inhibition (**Figures 2A, B**). Colony formation assay found that Agrin deficiency decreased clonogenic capability of NSCLC cells, and that PI3K/AKT activators partially rescued this capability (**Figures 2C**, **S2A, S4A**). Moreover, Agrin knockdown suppressed NSCLC cell proliferation, while SC79 reversed these effects (**Figures 2D**, **S2B**). Immunofluorescence illustrated that Agrin downregulation decreased the numbers of Ki67 positive cells, and that SC79 partially restored the decrease (**Figures 2E**, **S2C, E**). Cell cycle assays showed that Agrin deficiency induced G0/G1 arrest, while SC79 reversed this arrest (**Figures 2F, G**, **S2D, F**). Immunoblotting results indicated that Agrin downregulation decreased CyclinD1 and CDK4/6 protein levels, and that PI3K/AKT activators rescued their expression (**Figures 2H**, **S2G, S4B**). These results suggested that Agrin deficiency suppressed NSCLC cell proliferation and induced cell cycle arrest *via* inhibiting PI3K/AKT pathway.

### Agrin Silencing Inhibits NSCLC Cell Migration and Invasion *via* Suppressing PI3K/AKT Pathway

**Figure 3:**
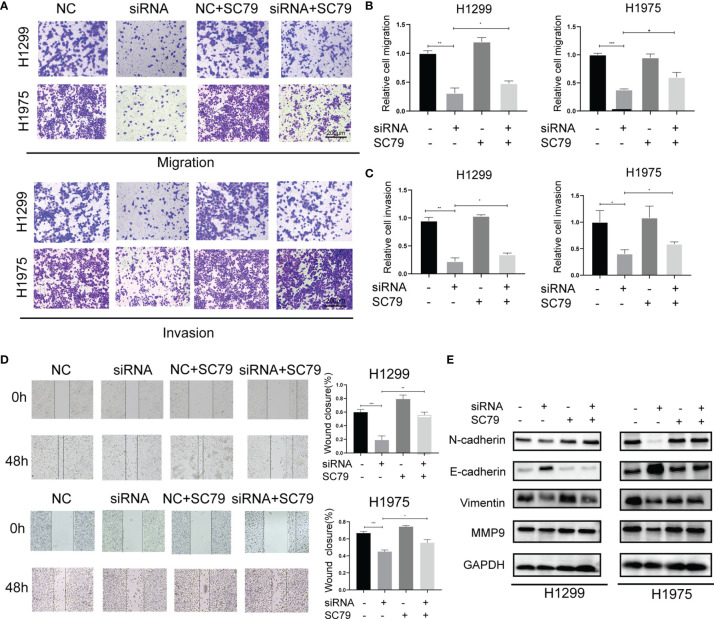
Agrin silencing inhibited NSCLC cell migration and invasion *via* suppressing PI3K/AKT pathway. **(A–C)** Representative images of modified Boyden chamber migration and invasion assays in NSCLC cells treated with Agrin siRNA and SC79. Scale bar, 200 μm. **(D)** Representative images of wound healing assays in NSCLC cells treated with Agrin siRNA and SC79. The images were taken at 100X magnification. Scale bar, 200 μm. **(E)** Representative immunoblots of N-Cadherin, E-Cadherin, Vimentin and MMP9. N = 3; *P < 0.05; **P < 0.01; ***P < 0.001.

In addition to cell proliferation, Agrin also regulated NSCLC cell metastasis. Modified Boyden chamber assay indicated that Agrin downregulation inhibited NSCLC cell migration and invasion, and that SC79 restored this inhibition (**Figures 3A**–**C**, **S3C**–**F**). Wound healing assay also suggested that depletion of Agrin significantly suppressed cell migration in both H1975 and H1299 cells, and that PI3K/AKT activators rescued this suppression (**Figures 3D**, **S3A, B, S4C**). In addition, lower levels of N-Cadherin, Vimentin, MMP9, and higher levels of E-Cadherin were detected by immunoblotting in the Agrin silencing group (**Figure S3G**). However, SC79 and 740Y-P reversed these effects (**Figures 3E**, **S4D**). These results suggested that Agrin deficiency suppressed NSCLC cell metastasis through PI3K/AKT pathway.

### Agrin Deficiency Suppresses Tregs *via* Downregulating PI3K/AKT/IL-6 Signaling Pathway

**Figure 4:**
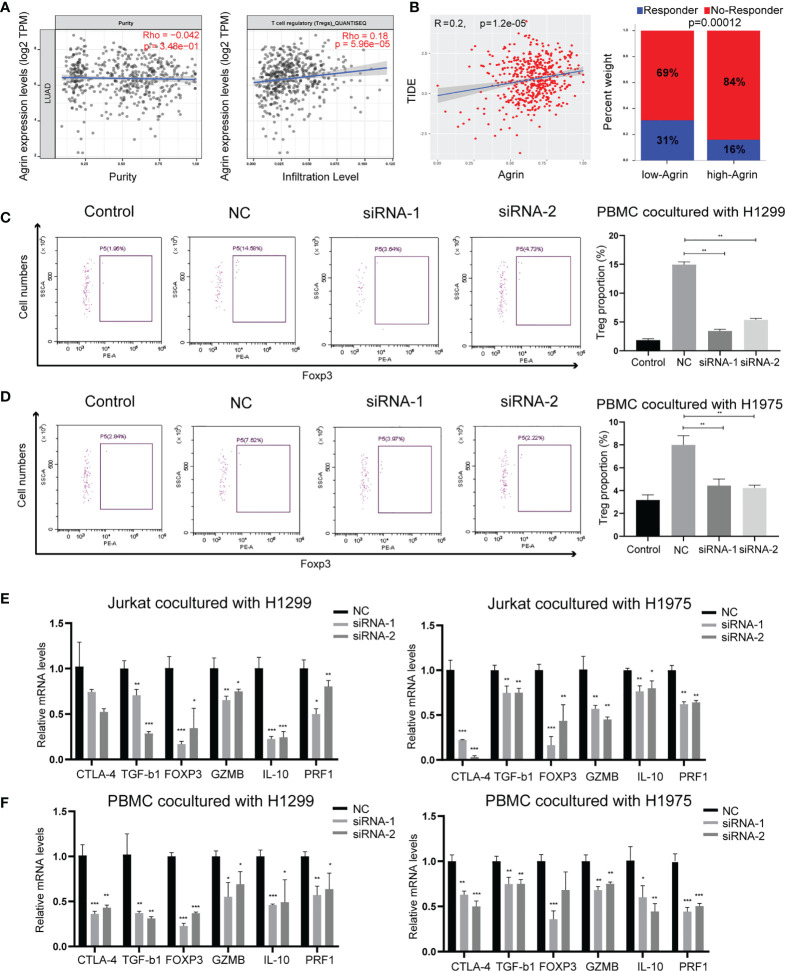
Agrin knockdown decreased Treg differentiation and downregulated Treg-related genes. **(A)** The relationship between Treg infiltration and Agrin expression in NSCLC from TIMER. **(B)** The immunotherapeutic response rates for NSCLC patients in TIDE between the low- and high-Agrin groups. **(C, D)** Flow cytometry of Tregs (CD4+, CD25+, Foxp3+) in PBMCs cocultured with NSCLC cells transfected with NC or siRNA. **(E, F)** Relative expression of Treg markers (CTLA-4, TGF-b1, Foxp3, GZMB, IL-10, PRF1) in Jurkat T cells and PBMCs cocultured with NSCLC cells transfected with NC or siRNA for 6 days. N = 3; *P < 0.05; **P < 0.01; ***P < 0.001.

**Figure 5:**
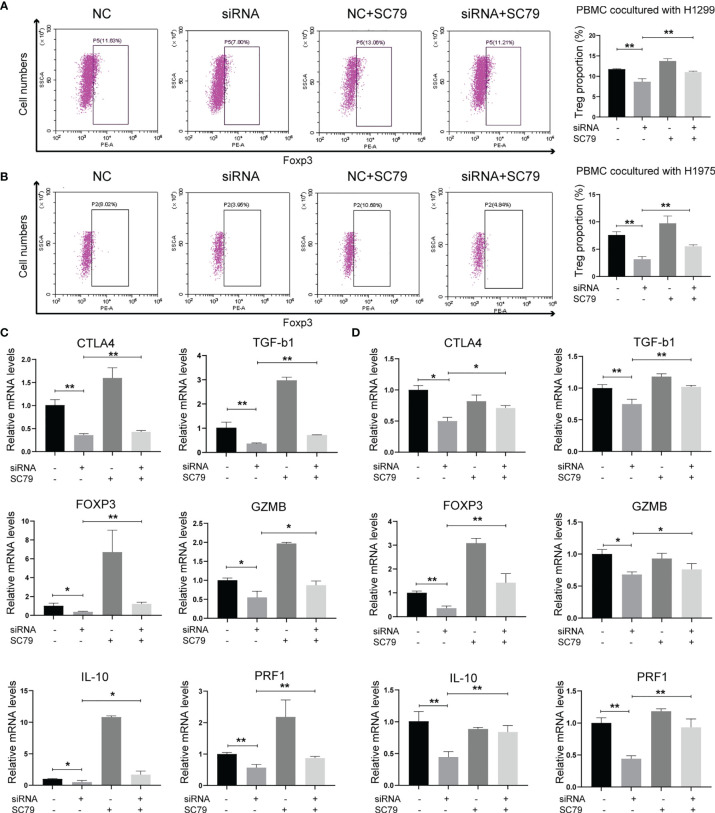
Agrin regulated Treg differentiation and Treg-related genes expression through PI3K/AKT pathway. **(A, B)** Flow cytometry of Tregs (CD4+, CD25+, Foxp3+) in PBMCs cocultured with NSCLC cells treated with Agrin siRNA and SC79. **(C, D)** Relative expression of Treg markers (CTLA-4, TGF-b1, Foxp3, GZMB, IL-10, PRF1) in Jurkat T cells and PBMCs cocultured NSCLC cells treated with Agrin siRNA and SC79 for 6 days. N = 3; *P < 0.05; **P < 0.01.

As Agrin was reported to be expressed in T cells and promote autoimmune disease development, we further explored the effects of Agrin on TIME using TIMER and TIDE website. The expression levels of Agrin were significantly associated with Treg infiltration and the response rates of immune checkpoint inhibitors in NSCLC patients (**Figures 4A, B**, **5A**). After coculturing with NSCLC cells for 6 days, the Treg proportions in PBMCs were significantly increased, while the percentages of Tregs were less in PBMCs cocultured with Agrin-deficient NSCLC cells (**Figures 4C, D**). The Treg-related genes (TGFB1, Foxp3, CTLA-4, IL-10, PRF1, GZMB) were declined in Jurkat T cells and PBMCs cocultured with Agrin-deficient NSCLC cells (**Figures 4E, F**).Although M1 macrophages and CD8 T cells also played an important role in TIME, no significant alteration of macrophage polarization or CD8 T cells was detected in the THP-1 cells or PBMCs cocultured with Agrin-deficient NSCLC cells (**Figures S5B, C**). Moreover, treatment with SC79 or 740Y-P in NSCLC cells could restore the proportions of Tregs in PBMCs and the Treg-related genes (**Figures 5**, **S4F**). There results revealed that Agrin silencing in NSCLC cells downregulated Tregs through PI3K/AKT signaling pathway.

**Figure 6:**
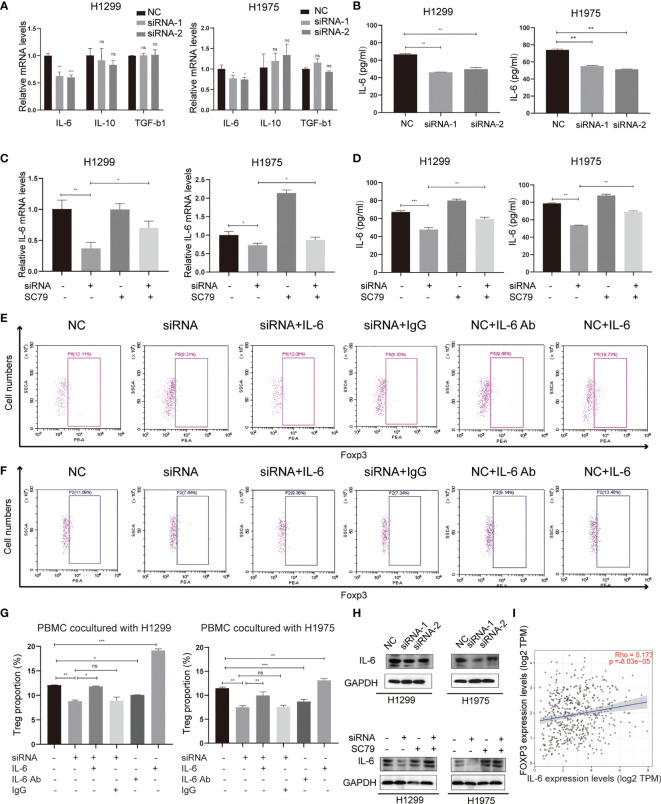
Agrin regulated Treg differentiation *via* modulating IL-6 expression and secretion. **(A)** Relative mRNA levels of IL-6, IL-10 and TGF-b1 in NSCLC cells transfected with NC or Agrin siRNA. **(B)** ELISA of IL-6 secretion levels in NSCLC cells transfected with NC or siRNA. **(C)** Relative expression of IL-6 in NSCLC cells treated with Agrin siRNA and SC79. **(D)** ELISA of IL-6 secretion levels in NSCLC cells treated with Agrin siRNA and SC79. **(E–G)** Flow cytometry of Tregs (CD4+, CD25+, Foxp3+) in PBMCs cocultured with NSCLC cells treated with Agrin siRNA and IL-6/IL-6 Ab. **(H)** Representative immunoblots of IL-6 in NSCLC cells treated with Agrin siRNA and SC79. **(I)** Correlation of IL-6 and Foxp3 expression in NSCLC (TIMER). N = 3; ns, nonsignificant; *P < 0.05; **P < 0.01; ***P < 0.001.

Several tumor-secreting cytokines, including IL-6, IL-10 and TGF-b1, are essential for Treg differentiation. To identify the mediators between NSCLC cells and Tregs, qPCR was used to screen these potential key cytokines in both H1299 and H1975 cells (**Figure 6A**). The results revealed that IL-6, rather than IL-10 and TGF-b1, was significantly downregulated in the Agrin-deficient NSCLC cells. ELISA confirmed the lower levels of IL-6 in the medium of Agrin-deficient NSCLC cells (**Figure 6B**). Moreover, the addition of PI3K/AKT activators reversed the decrease of IL-6 mRNA levels in NSCLC cells and protein levels in the medium (**Figures 6C, D**). In addition, exogenous IL-6 treatment restored Treg proportions in PBMCs cocultured with Agrin-deficient NSCLC cells, while the addition of IL-6 antibodies significantly decreased the percentages of Tregs in PBMCs (**Figures 6E**–**G**). The results of immunoblotting indicated that Agrin silencing downregulated IL-6 in NSCLC cells, and that SC79 or 740Y-P could reverse these effects (**Figures 6H**, **S4E**). TIMER website revealed that IL-6 and Foxp3 were closely associated in NSCLC cells (**Figure 6I**). Collectively, these observations indicated that Agrin knockdown inhibited Treg differentiation *via* downregulating PI3K/AKT/IL-6 pathway.

### Agrin Knockdown Alleviates NSCLC Cell Growth and Treg Infiltration *In Vivo*

**Figure 7:**
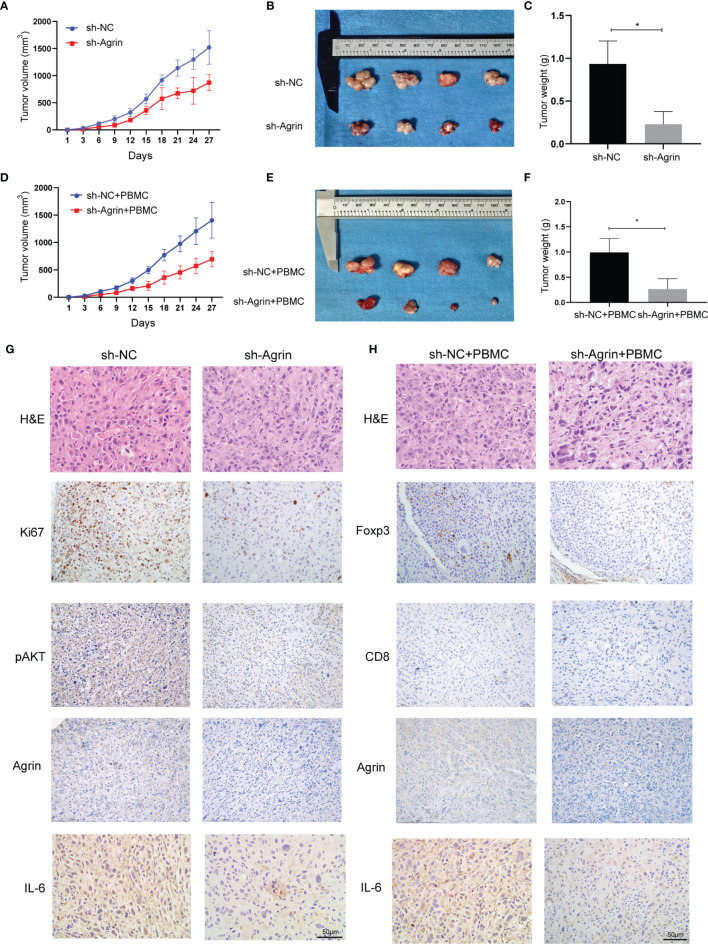
Agrin silencing prohibited NSCLC cell growth, and alleviated IL-6, p-AKT and Foxp3 expression *in vivo*. **(A–F)** Agrin knockdown suppressed NSCLC cell growth in the xenograft tumor model of BALB/C nude mice with or without PBMC injection. **(G, H)** Representative images of Ki67, Foxp3, p-AKT, CD8, Agrin and IL-6 staining in the tumor tissues. The images were taken at 200X magnification. Scale bar, 50 μm. N ≥ 4; *P < 0.05.

To investigate the effects of Agrin downregulation on NSCLC cell growth and Treg infiltration *in vivo*, Agrin was downregulated with lentiviral-mediated shRNAs. After puromycin selection, both the transcriptional and protein levels of Agrin were significantly decreased (**Figure S1D**). In the xenograft tumor model using BALB/C nude mice, Agrin deficiency significantly suppressed NSCLC cell growth *in vivo* (**Figures 7A**–**C**). In the group of PBMC injection, Agrin knockdown also inhibited tumor growth (**Figures 7D**–**F**). The IHC results indicated that Agrin knockdown inhibited NSCLC cell proliferation, IL-6 and p-AKT expression *in vivo* (**Figure 7G**). Moreover, Agrin deficiency decreased the expression levels of Foxp3 and IL-6 in NSCLC TIME (**Figure 7H**). Taken together, our results suggested that Agrin downregulation significantly suppressed NSCLC cell growth and Treg infiltration *in vivo*.

## Discussion

NSCLC is the most lethal malignancy worldwide. Due to its asymptomatic and rapid progression, most patients were diagnosed at advanced stages and had poor prognosis. Immunotherapy is a high-profile therapy by largely extending patients’ survival time based on enriched immune-infiltrated environment in NSCLC (18–21). However, a considerable number of patients could not benefit from immunotherapy. Recent studies illustrated that immune suppressive environment in NSCLC might hinder patients from getting profits from immune checkpoint inhibitors (22). Tumor progression has an inextricable association with TIME (23). Cancer cells not only enhance several oncogenic signaling pathways to promote tumor progression, but also secrete cytokines to stimulate and activate immune-suppressive cells, such as Tregs, tumor-associated fibroblasts and cancer stem cells, which in turn assist tumor to evade immune surveillance (24–29). Tregs repress tumor killing T cell responses (30), as the main impediments of cancer immunotherapy. Clinical researches approved that Treg exhaustion led to tumor regression (31) and induced anti-cancer immunity (32). Therefore, finding a way to inhibit tumor development and depleting Tregs might be effective to improve NSCLC prognosis.

Agrin exerts its vital roles in neuro synapse formation (5), auto-immune disease (13), angiogenesis (33) and various cancer development (6–9). In addition, Agrin is a key mediator in primary T cell activation in autoimmune disease (11–13). However, few study associated Agrin with TIME. In our study, samples from TCGA dataset suggested that Agrin was highly expressed in NSCLC compared with normal lung tissues and associated with worse survival. Data of qRT-PCR confirmed upregulated Agrin levels in NSCLC compared with the adjacent normal lung tissues. Subsequent experiments indicated that Agrin deficiency inhibited NSCLC cell proliferation and invasion. PI3K/AKT pathway, a classical oncogenic signaling pathway, could facilitate multiple tumor amplification *via* increasing cell viability (34, 35). Intracellular PI3K/AKT pathway is important for mediating epithelial-mesenchymal transition (EMT) *via* inhibiting E-cadherin transcription (36), inducing N-cadherin (37) and other key EMT drivers (such as Snail and Slug). Our enriched analysis showed that Agrin was correlated with PI3K/pAKT signaling pathway. Following studies indicated that PI3K/AKT activators mitigated the effects of Agrin downregulation on NSCLC cells. Previous studies have shown that agrin can phosphorylate and activate Src kinase (38). Src/PI3K/AKT axis plays important roles in the development and drug resistance of various tumors (39–44). In lung cancer, Src/Akt pathway can promote the invasion and metastasis of lung cancer cells, suggesting that Src kinase may be an important factor for Agrin in regulating PI3K/AKT signal and causing tumor development (41, 42, 45, 46). In addition, Wang et al. reported that Agrin in rectal cancer induced WNT pathway to promote rectal cancer development (47). Our results also verified WNT pathway-related proteins (c-myc, GSK-3β, β-cantein) were downregulated in the Agrin-deficient groups (**Figure S2G**). Glycogen synthase kinase-3β (GSK-3β) is a key protein in WNT pathway, also known as a tumor inhibitor. AKT could phosphorylate and inactivate GSK-3β at Ser9 (48, 49), which subsequently stabilized Snail protein and finally caused EMT (50, 51). Consistently, accumulated evidence revealed that activation of PI3K/AKT/GSK-3β pathway resulted in lung cancer progression (52), hepatocellular carcinoma metastasis (53) and so on. These data suggested that Agrin might exert its pro-oncogenic function *via* PI3K/AKT pathway, with GSK-3β as a potential downstream target.

Based on the reports of Agrin in auto-immune diseases and T cells, we further explored the influence of Agrin on T cells in NSCLC TIME. Agrin was positively correlated with Treg infiltration and negatively associated with the response of immune checkpoint inhibitors in NSCLC patients. After coculture with NSCLC cells, the proportion of Tregs (CD4+, CD25+, Foxp3+) in PBMCs was decreased in the Agrin-deficient group. Tregs exert immunosuppressive mechanism in 2 ways: secret cytokines and direct contact (22). Treg-produced immunosuppressive cytokines (TGF-b1, IL-10) restrain the function of T effector cells (54–56). The cytokines secreted from Tregs, such as perforin and granzyme B, could directly kill anti-cancer immune cells, including antigen presenting cells and T effector cells. Moreover, immune checkpoint CTLA-4 on the surface of Tregs bind to their targets on anti-cancer immune cells, and inhibit their functions. A key transcription factor for Treg function is Foxp3, forced expression of which coverts naïve T cells into immunosuppressive Treg-like cells (57). These studies indicated that Treg-related cytokines and immune biomarker might be important for the immunosuppressive functions of Tregs. In the present work, Treg-related mRNAs were downregulated in Jurkat T cells and PBMCs after cocultured with Agrin-deficient NSCLC cells, suggesting that knocking down Agrin in NSCLC cells declined Treg differentiation in TIME. Based on the findings that PI3K/AKT pathway participated in Agrin-facilitating NSCLC progression, we assumed that PI3K/AKT pathway might be a pivotal factor for Agrin-promoting Treg differentiation. As expected, the decreased Treg differentiation was abrogated after the addition of PI3K/AKT activators in the Agrin-deficient NSCLC cells.

Various cytokines are involved in the process of Treg differentiation, including IL-6, IL-10 and TGF-b1 (58). Agrin downregulation suppressed IL-6 production, followed by the decrease of Treg differentiation. IL-6 was reported to mediate various solid cancer behaviors, such as tumor growth and metastasis (59–62). Moreover, IL-6 is associated with poor prognosis of lung cancer patients (63). In addition, patients with elevated levels of circulating IL-6 are more likely to have higher Treg proportions and resistance to treatments (64–66), implicating that blocking IL-6 might result in therapeutic gain of NSCLC therapy. However, little research was concentrated on its roles in TIME. In our study, we first demonstrated that IL-6 was a critical mediator for Agrin to facilitate Treg infiltration in NSCLC. Based on the tumor-promoting characteristic of PI3K/AKT pathway and previous studies on AKT-regulating IL-6 expression, we subsequently assessed the effects of PI3K/AKT activators on IL-6 levels. From qPCR, immunoblotting and ELISA, we found that SC79 restored the expression and secretion of IL-6 in Agrin-deficient NSCLCs. These results implicated that PI3K/pAKT pathway was necessary for Agrin-stimulated IL-6 production.

Due to the lack of immune competence, our nude mouse model insufficiently simulated complex immune regulatory responses in tumor microenvironments *in vivo*. Further investigation on function of Agrin in immune-competent mice would be required in future studies.

## Conclusions

**Figure 8:**
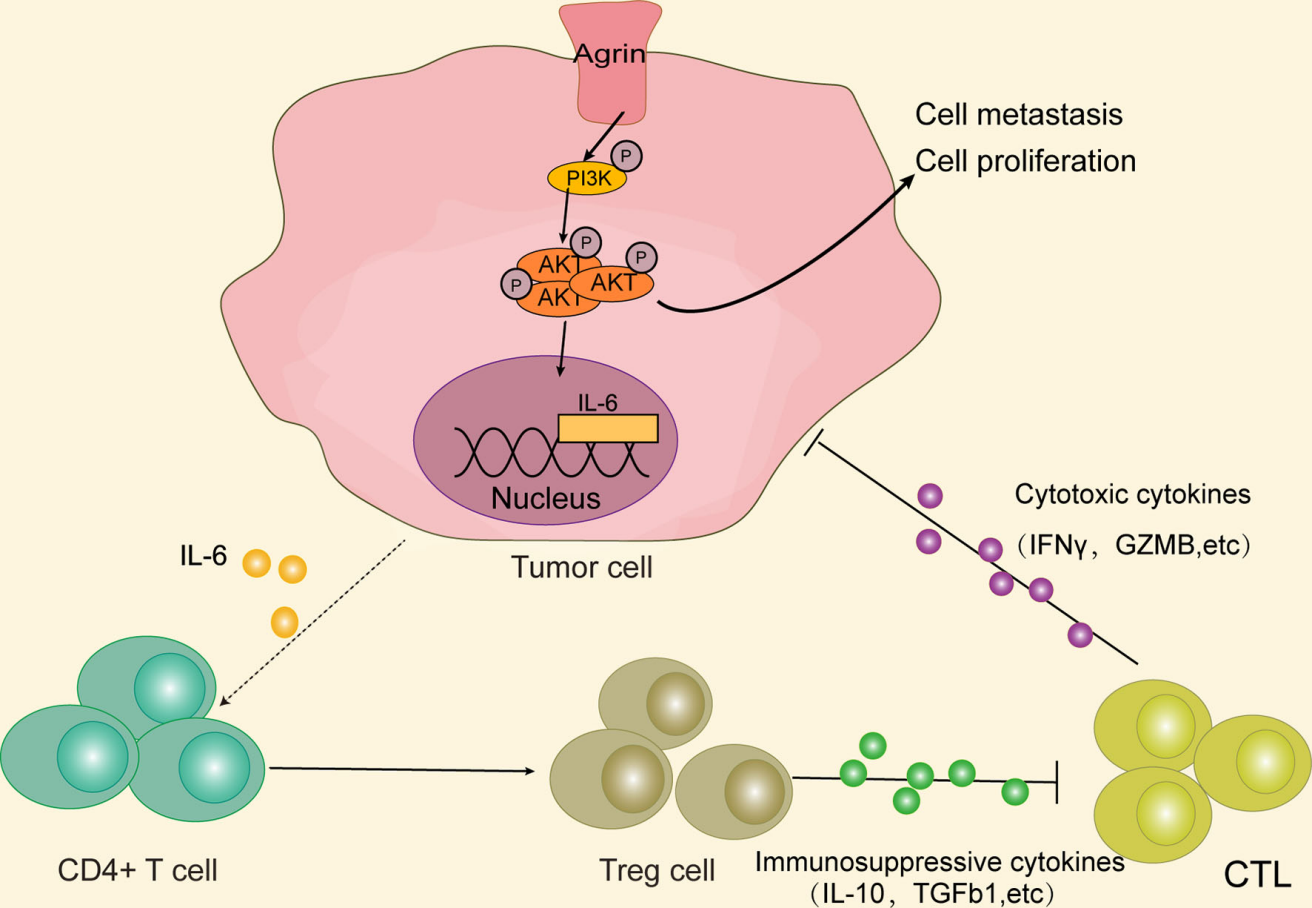
Agrin promotes NSCLC progression and Treg differentiation *via* inducing PI3K/AKT signaling pathway. Agrin promotes NSCLC cell proliferation and metastasis, as well as IL-6 expression and secretion, *via* stimulating PI3K/AKT signaling pathway. IL-6 subsequently induces Treg differentiation. Immunosuppressive cytokines secreted by Tregs might inhibit cytotoxic T cells from releasing cytotoxic cytokines, ultimately triggering tumor immune evasion.

In summary, our study originally indicated that higher Agrin levels were associated with worse survival in NSCLC patients. Agrin enhanced tumor growth and invasion through PI3K/AKT pathway, and induced IL-6 expression and secretion in NSCLC cells to promote Treg differentiation. Therefore, Agrin/PI3K/AKT/IL-6 axis might be a novel potential target for NSCLC therapies (**Figure 8**).

## Data Availability Statement

The original contributions presented in the study are included in the article/**Supplementary Material**. Further inquiries can be directed to the corresponding authors.

## Ethics Statement

The studies involving human participants were reviewed and approved by The Institutional Review Board at Zhongnan Hospital of Wuhan University. The patients/participants provided their written informed consent to participate in this study. The animal study was reviewed and approved by The Institutional Animal Care and Use Committee at Zhongnan Hospital of Wuhan University.

## Author Contributions

LH, CX, and YGong designed the study. LH, HS, SM, YL, and WS acquired and interpretated the clinical and preclinical data. SL, NZ, XJ, YGao, and ZH collected and analyzed the *in vitro* and *in vivo* data. All authors read and approved the final manuscript.

## Funding

This study was supported by National Natural Science Foundation of China (81800429, 81773236 and 81972852), Key Research & Development Project of Hubei Province (2020BCA069), Nature Science Foundation of Hubei Province (2020CFB612), Health Commission of Hubei Province Medical Leading Talent Project, Young and Middle-Aged Medical Backbone Talents of Wuhan (WHQG201902), Application Foundation Frontier Project of Wuhan (2020020601012221), Zhongnan Hospital of Wuhan University Science, Technology and Innovation Seed Fund (znpy2019001 and znpy2019048), and Translational Medicine and Interdisciplinary Research Joint Fund of Zhongnan Hospital of Wuhan University (ZNJC201922 and ZNJC202007).

## Conflict of Interest

The authors declare that the research was conducted in the absence of any commercial or financial relationships that could be construed as a potential conflict of interest.

## Publisher’s Note

All claims expressed in this article are solely those of the authors and do not necessarily represent those of their affiliated organizations, or those of the publisher, the editors and the reviewers. Any product that may be evaluated in this article, or claim that may be made by its manufacturer, is not guaranteed or endorsed by the publisher.
