# Supplementary material for: Agrin Promotes Non-Small Cell Lung Cancer Progression and Stimulates Regulatory T Cells via Increasing IL-6 Secretion Through PI3K/AKT Pathway
Source: Front Oncol. 2022 Jan 17;11:804418. doi: 10.3389/fonc.2021.804418 (PMC8801576; doi:10.3389/fonc.2021.804418)
Supplement: Supplementary file 1 [file DataSheet_1.pdf]

Table S1 | Antibody information.

Antibody	Dilution	Company
Agrin (A17320)	1:1000	Abclonal
Agrin (160842)	1:100	ZEN BIO
AKT (4691)	1:1000	Cell Signaling Technology
CD8 (ab217344)	1:2000	Abcam
CDK6 (14052-1-AP)	1:1000	Proteintech
CDK4 (11026-1-AP)	1:1000	Proteintech
CDK2 (10122-1-AP)	1:1000	Proteintech
CyclinD1 (26939–1-AP)	1:1000	Proteintech
CyclinE1 (11554-1-AP)	1:1000	Proteintech
E-Cadherin (A3044)	1:1000	Abclonal
Foxp3 (ab20034)	1:1000	Abcam
GAPDH (10494-1-AP)	1:5000	Proteintech
IL-6 (A0286)	1:1000	Abclonal
Ki67 (27309-1-AP)	1:200	Abcam
MMP9 (A2095)	1:1000	Abclonal
N-Cadherin (A19083)	1:1000	Abclonal
pAKT (4060)	1:1000	Cell Signaling Technology
pAKT (AP0140)	1:100	Abclonal
Vimentin (A19607)	1:1000	Abclonal

Table S2 | qPCR primer sequences.

Gene	sequences or target sequence (5' - 3')
Agrin-F	ACGGAGTCACCTACGAAAACG
Agrin-R	AGCAATCACTGTCATACGTGC
CTLA4-F	AGGTGACTGAAGTCTGTGCG
CTLA4-R	CATGAGCTCCACCTTGCAGA
FOXP3-F	TACCACAATATGCGACCCCC
FOXP3-R	GGCGAACATGCGAGTAAACC
GAPDH-F	CATCATCCCTGCCTCTACTGG
GAPDH-R	GTGGGTGTCGCTGTTGAAGTC
GZMB-F	CAGCTGGAGAGAAAGGCCAA
GZMB-R	TGGCGTAAGTCAGATTCGCA
IL-10-F	AAGACCCAGACATCAAGGCG
IL-10-R	AGGCATTCTTCACCTGCTCC
IL-6-F	GCCGCATCGCCGTCTCCTAC
IL-6-R	CCTCAGCCCCCTCTGGGGTC
PRF1-F	TCCTAAGCCCACCAGCAATG
PRF1-R	AAGGAGGCCGTCATCTTGTG
TGF-b1-F	ACATTGACTTCCGCAAGGAC
TGF-b1-R	CCGGGTTATGCTGGTTGTA

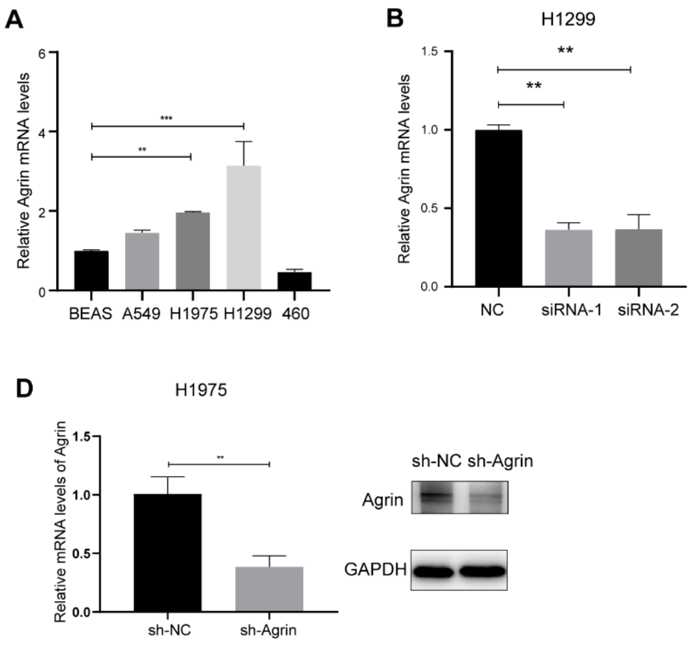

Figure S1 | The expression and downregulation of Agrin in NSCLC cells. **(A)** The mRNA levels of Agrin in NSCLC and lung epithelial cells. **(B, C)** Verification of Agrin knockdown efficiency in H1299 and H1975 cells. **(D)** Stable knockdown of Agrin in H1975 cells. Relative Agrin mRNA levels in sh-NC and sh-Agrin H1975 cells (left)**,** representative immunoblots of Agrin in sh-NC and sh-Agrin H1975 cells. (right). N = 3; **, $p < 0.01$; ***, $p < 0.001$.

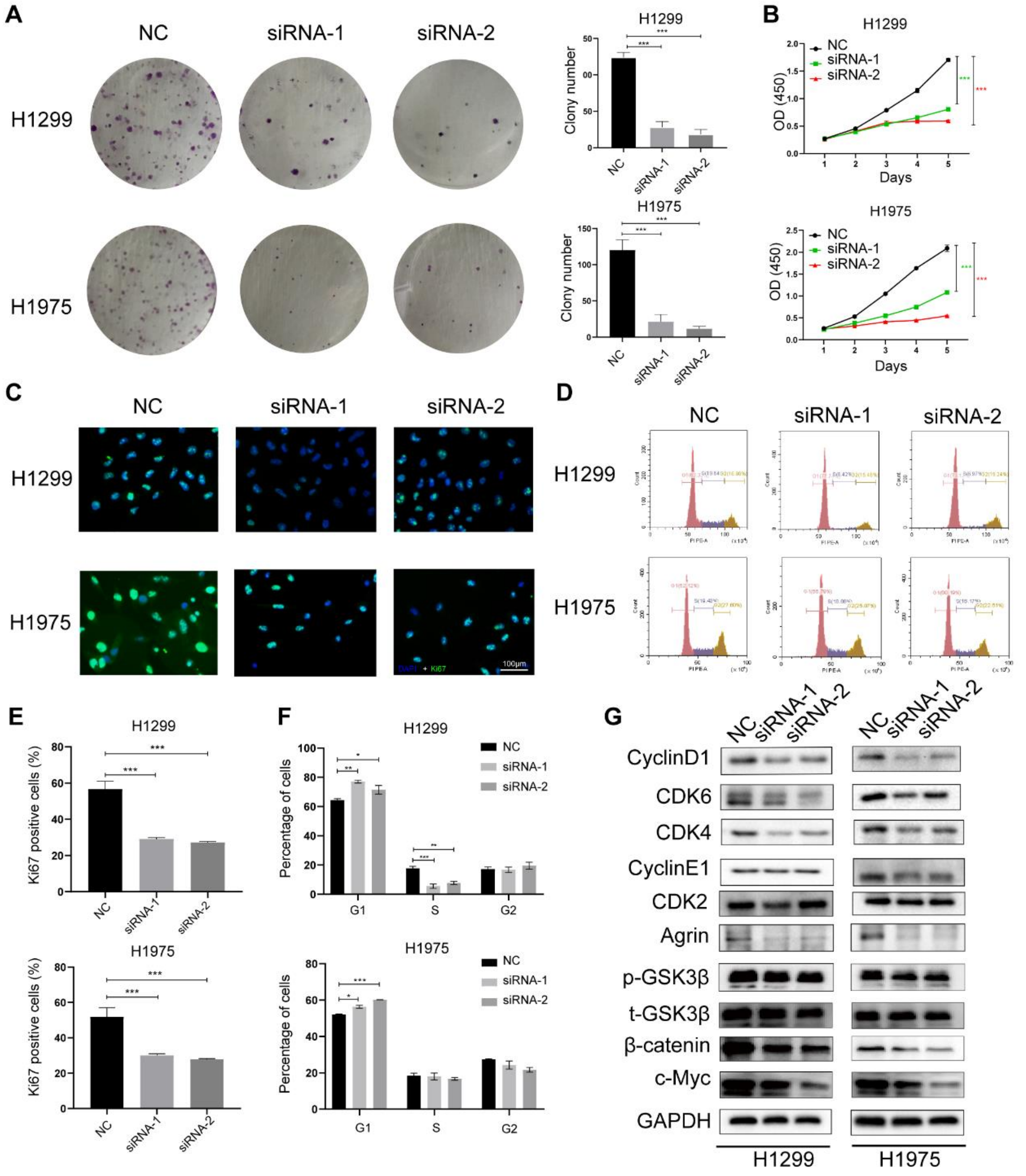

Figure S2 | Agrin knockdown inhibited NSCLC cell proliferation and induced cell cycle arrest. **(A)** Colony formation assay was used to assess the proliferative ability of NSCLC cells transfected with NC or siRNA. **(B)** OD values of CCK-8 assay in NSCLC cells transfected with NC or siRNA. **(C, E)** Representative images of Ki-67 staining in NSCLC cells transfected with NC or siRNA. The images were taken at 400X magnification. Scale bar, 100 μm. **(D, F)** Flow cytometry of cell cycle in NSCLC cells transfected with NC or siRNA. **(G)** Representative immunoblots of Agrin, Cyclin D1, Cyclin E1, CDK2/4/6, p-GSK3β, t-GSK3β, β-catenin and c-myc proteins. N = 3; *, P < 0.05, **, P < 0.01, ***, P < 0.001.

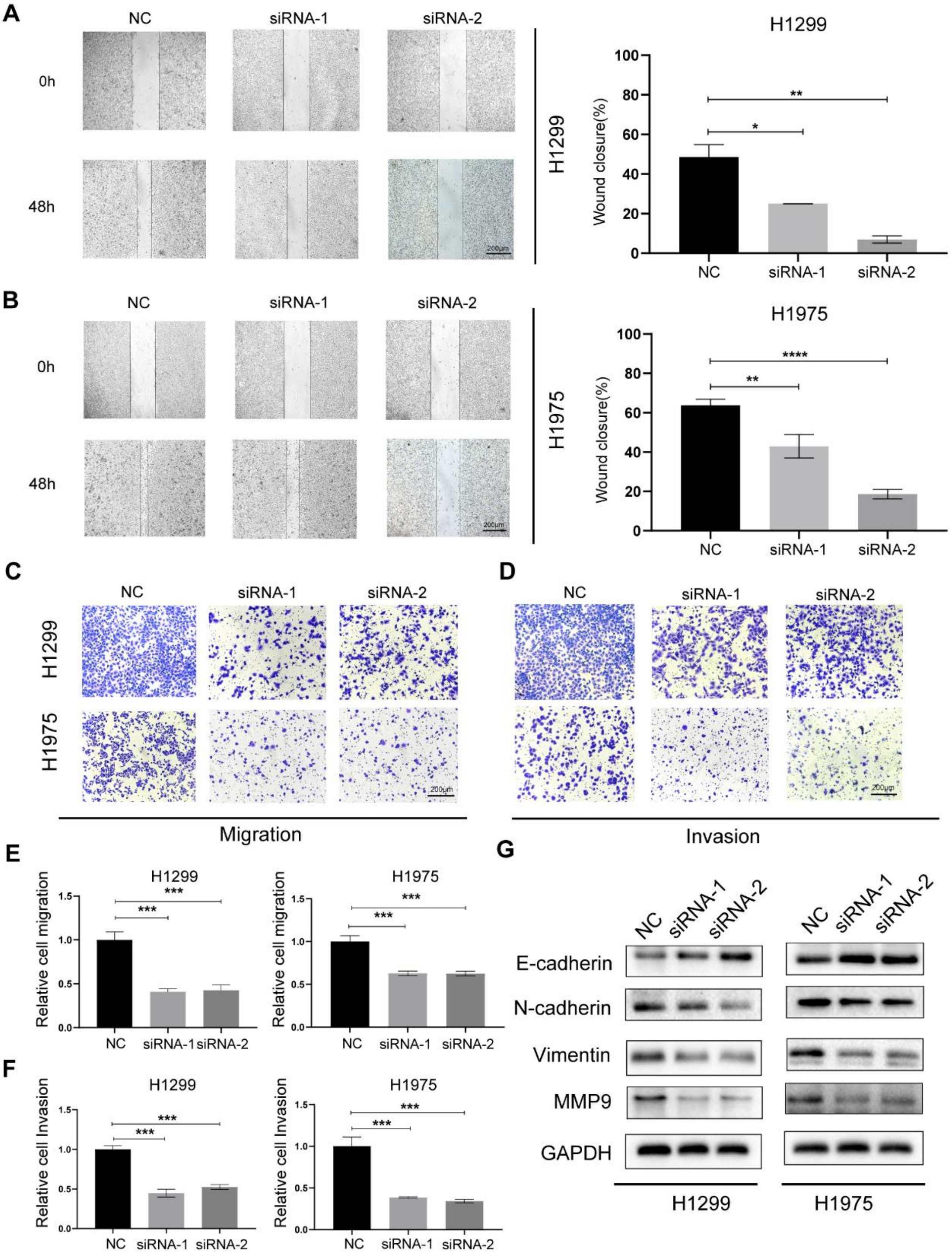

Figure S3 | Agrin knockdown inhibited NSCLC cell migration and invasion. **(A, B)** Wound healing assay of NSCLC cells transfected with NC or siRNA. The images were taken at 100X magnification. Scale bar, 200 μm. **(C-F)** Modified Boyden chamber migration and invasion assay of NSCLC cells transfected with NC or siRNA. Scale bar, 200 μm. **(G)** Representative immunoblots of N-Cadherin, E-Cadherin, Vimentin and MMP9. N = 3; *, $P < 0.05$; **, $P < 0.01$; ***, $P < 0.001$.

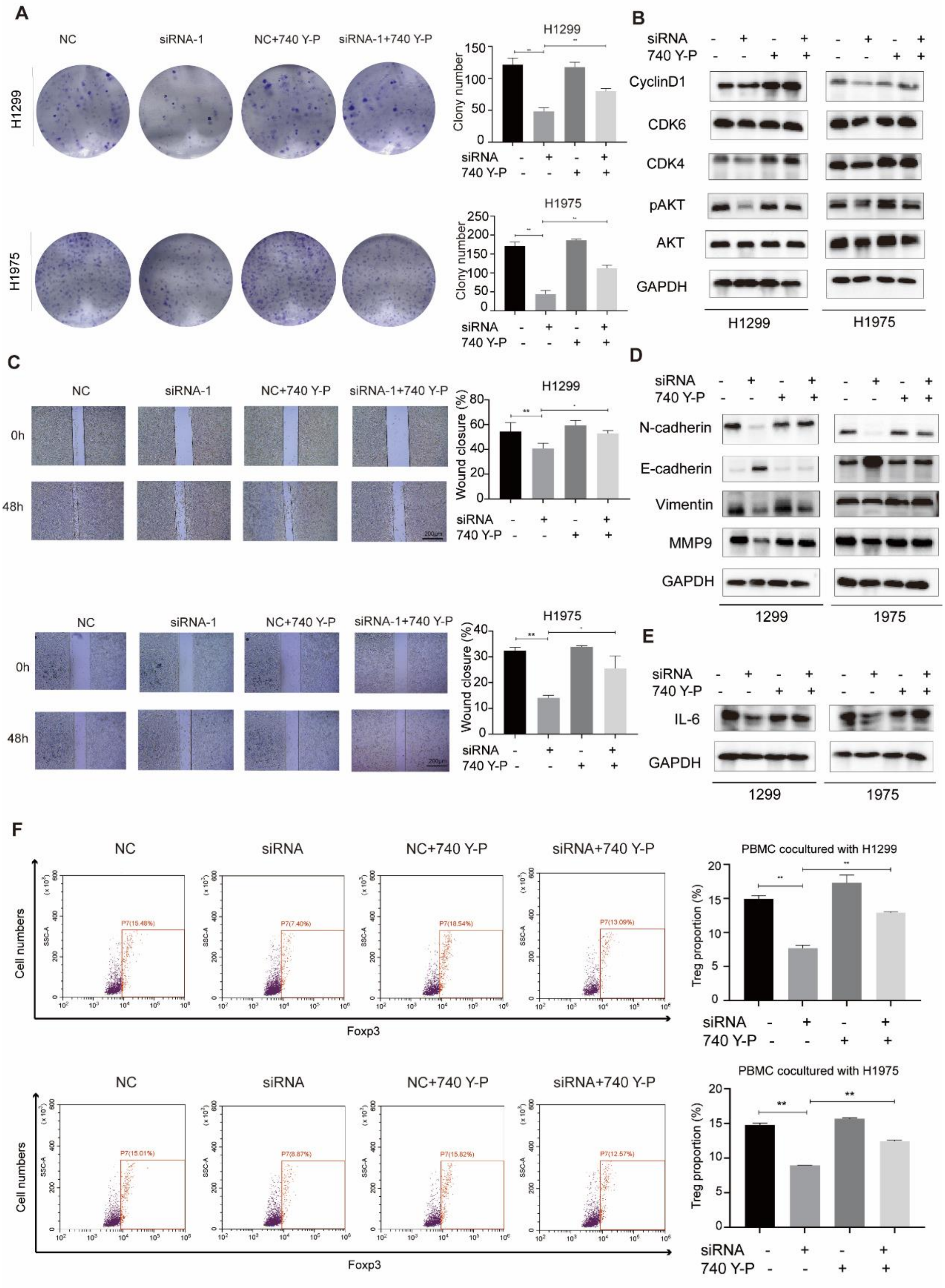

Figure S4 | PI3K activator 740Y-P rescued NSCLC cell proliferation, migration and Treg cell differentiation in the Agrin-deficient groups. **(A)** 740Y-P (25 µg/ml) rescued colony formation inhibited by Agrin deficiency in NSCLC cells. **(B)** Representative immunoblots of Cyclin D1, CDK4/6, pAKT and AKT in NSCLC cells treated with Agrin siRNA and 740Y-P. **(C)** 740Y-P rescued NSCLC cell migration inhibited by Agrin deficiency. **(D)** Representative immunoblots of N-Cadherin, E-Cadherin, Vimentin and MMP9 in NSCLC cells treated with Agrin siRNA and 740Y-P. **(E)** Representative immunoblots of IL-6 in NSCLC cells treated with Agrin siRNA and

740Y-P. **(F)** Flow cytometry of Tregs (CD4+, CD25+, Foxp3+) in PBMCs cocultured with NSCLC cells treated with Agrin siRNA and 740Y-P. N = 3; *, P < 0.05; **, P < 0.01.

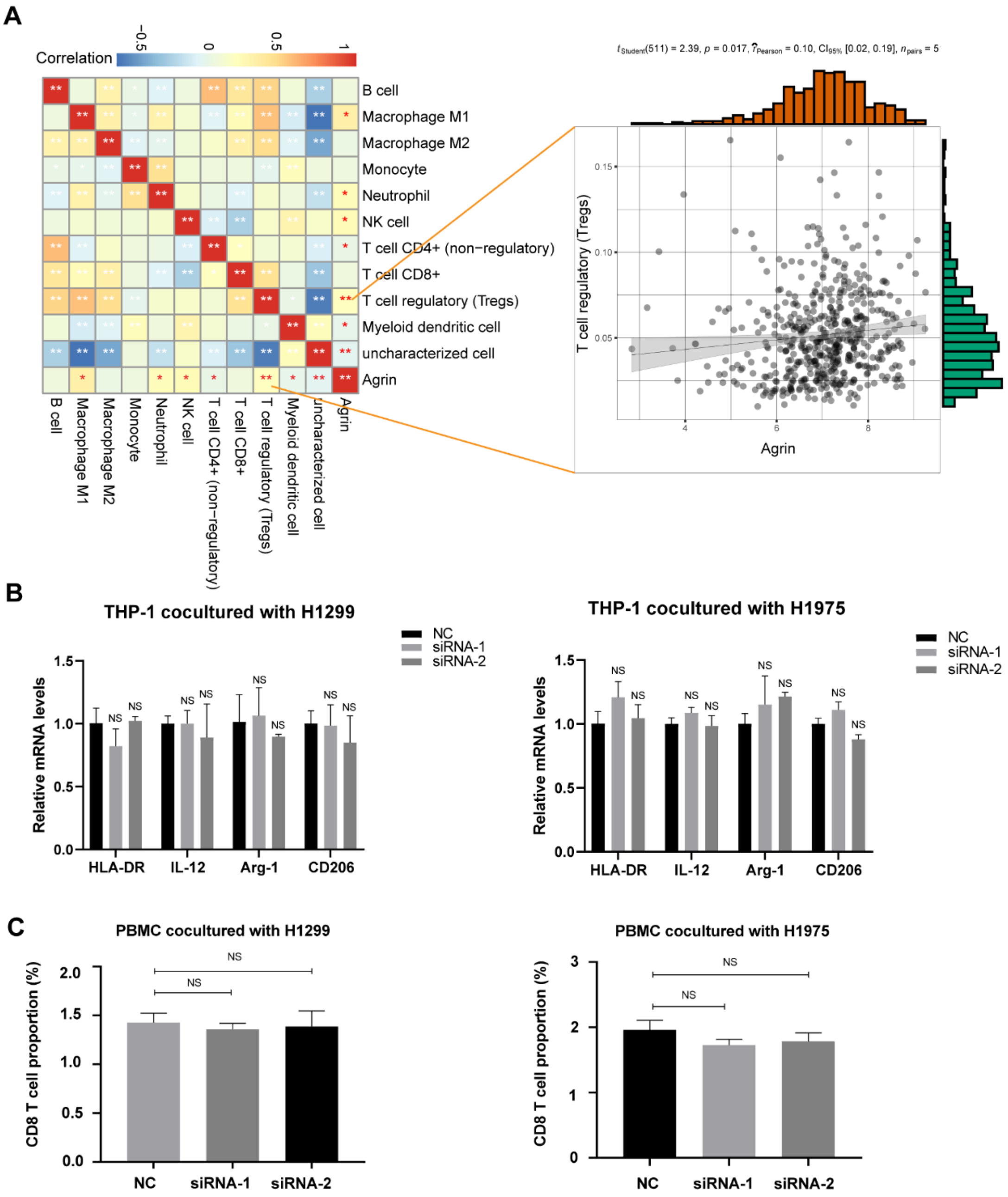

Supplementary Figure 5 | Correlation between immune cells infiltration and Agrin expression. **(A)** QUANTISEQ algorithm was used to analyze the correlation between immune cell infiltration and Agrin expression. **(B)** Relative expression of macrophage polarization markers (M1: HLA-DR, IL-12; M2: Arg-1, CD206) in THP-1 cells cocultured with NSCLC cells transfected with NC or siRNA. **(C)** Flow cytometry of CD8 T cells in PBMCs cocultured with NSCLC cells treated with NC, or siRNA. N = 3; NS, not significant.
